# Growing rod analysis for the fusionless correction of Early Onset Scoliosis (EOS)

**DOI:** 10.1186/1748-7161-10-S1-O24

**Published:** 2015-01-19

**Authors:** Mark E Quick, Mark J Pearcy, Clayton J Adams, Geoffrey N Askin, Robert D Labrom

**Affiliations:** 1 QUT/Mater Paediatric Spine Research Group, Institute of Health and Biomedical Innovation, Queensland University of Technology and Mater Health Services, Brisbane, Australia

## Introduction

Managing spinal deformities in young children is challenging, particularly early onset scoliosis (EOS). Surgical intervention is often required if EOS has been unresponsive to conservative treatment particularly with rapidly progressive curves. An emerging treatment option for EOS is fusionless scoliosis surgery. Similar to bracing, this surgical option potentially harnesses growth, motion and function of the spine along with correcting spinal deformity. Dual growing rods are one such fusionless treatment, which aims to modulate growth of the vertebrae. The aim of this study was to ascertain the extent to which semi-constrained growing rods (Medtronic) with a telescopic sleeve component, reduce rotational constraint on the spine compared with standard rigid rods and hence potentially provide a more physiological mechanical environment for the growing spine.

## Methods

Six 40-60kg English Large White porcine spines served as a model for the paediatric human spine. Each spine was dissected into a 7 level thoracolumbar multi-segment unit (MSU), removing all non-ligamentous soft tissues and leaving 3cm of ribs either side. Pure nondestructive axial rotation moments of +/-4Nm at a constant rotation rate of 8deg/s were applied to the mounted MSU spines using a biaxial Instron testing machine. Displacement of each vertebral level was captured using a 3D motion tracking system (Optotrak). Each spine was tested in an un-instrumented state first and then with appropriately sized semi-constrained growing rods and rigid rods in alternating sequence. The rods were secured by multi-axial pedicle screws (Medtronic) at levels 2 and 6 of the construct. The range of motion (ROM), neutral zone (NZ) size and stiffness (Nm/deg) were calculated from the Instron load-displacement data and intervertebral ROM was calculated through a MATLAB algorithm from Optotrak data.

## Results

Irrespective of the order of testing, rigid rods significantly reduced the total ROM compared with semi-constrained rods (p<0.05) which resulted in a significantly stiffer spine for both left and right axial rotation (p<0.05). Analysing the intervertebral motion within the instrumented levels 2-6, rigid rods showed reduced ROM compared with semi-constrained growing rods and compared with un-instrumented motion segments.

## Conclusion

Semi-constrained growing rods maintain similar stiffness in axial rotation to un-instrumented spines, while dual rigid rods significantly reduce axial rotation. Clinically the effect of semi-constrained growing rods as observed in this study is that they would be expected to allow growth via the telescopic rod components while maintaining the axial flexibility of the spine and improved capacity for final correction.

